# Thermotolerance elicits specific genes in cucurbit plants as a response to the combined effect of viral infection and temperature stress

**DOI:** 10.1093/jxb/eraf277

**Published:** 2025-06-24

**Authors:** Celia de Moya-Ruiz, Pedro Gómez

**Affiliations:** Departamento de Biología del Estrés y Patología Vegetal, Centro de Edafología y Biología Aplicada del Segura (CEBAS)-CSIC, PO Box 164, Murcia 30100, Spain; Departamento de Biología del Estrés y Patología Vegetal, Centro de Edafología y Biología Aplicada del Segura (CEBAS)-CSIC, PO Box 164, Murcia 30100, Spain; University of Ghent, Belgium

**Keywords:** Cucurbit infection, heat stress, plant resilience, plant–virus–environment interaction, temperature, WMV

## Abstract

Plants respond to biotic and abiotic factors through specific physiological and metabolic changes that optimize their survival. However, the extent to which the combination of both stressors could modulate common or exclusive signaling pathways remains unclear, as most studies on gene-specific plant expression focus on a single stress. In this study, we examined the gene expression patterns in two cucurbit species, melon and zucchini, each with high- and low-temperature-tolerant cultivars, under watermelon mosaic virus (WMV) infection and three temperature ranges (20/14, 26/20, and 32/24 °C). Our results showed that WMV accumulation was higher in zucchini than in melon plants and was influenced by both thermotolerance and temperature conditions. Comparative 3′ mRNA-seq analysis revealed that zucchini exhibited a 2.6-fold higher percentage of differentially expressed genes (DEGs) than melon, with the highest percentage of DEGs occurring at lower temperatures in the thermosusceptible plants of both species, possibly associated with greater WMV accumulation. Notably, the greatest number of unique DEGs was detected in high-temperature and WMV-infected plants of thermotolerant melon (711 DEGs) and thermosusceptible zucchini (306 DEGs). Among the top 15 Gene Ontology terms, four orthologous genes were identified, three of which—MELO3C023308, MELO3C024920, and Cp4.1LG05g12560—exhibited a significant temperature-dependent expression under WMV infection, and potentially encode an F-box protein, a metal ion transporter, and a photomorphogenesis-related factor, respectively. These findings provide novel insights into plant–virus–environment interactions and may contribute to enhancing cucurbit crop resilience and food security.

## Teaser

Thermotolerant and thermosensitive cultivars of melon and zucchini plants exhibit differentially expressed genes that respond to the combination of watermelon mosaic virus infection and temperature stress.

## Introduction

Plants must adapt to a myriad of abiotic and biotic stresses to thrive. Extreme temperatures, drought, salinity, and greenhouse gases can limit plant growth by triggering specific physiological processes and metabolic responses that lead to gene-specific changes in plant development (Mittler *et al.*, 2012; Bailey-Serres *et al.*, 2019; Zhang *et al.*, 2022). Consequently, climate change poses a significant threat to global food production by affecting agricultural yield, food quality, and prices (Vermeulen *et al.*, 2012; Raza *et al.*, 2019; Thiault *et al.*, 2019). As climate change intensifies, crops face the impact of pests and pathogens, which can further limit food production and exacerbate food insecurity (Oerke, 2006; Pandey *et al.*, 2017; Rudgers *et al.*, 2020; Montes and Pagán, 2022; Tonnang *et al.*, 2022; Vasquez *et al.*, 2022; Xiao *et al*., 2024; Laine, 2023). Thus, understanding the intricate interactions between abiotic and biotic stressors is essential to mitigate the potential consequences on agricultural food production systems and to further adapt crop management practices to protect food production (Shelake *et al.*, 2024).

Heat stress can cause crop production losses by directly interfering with plant physiological processes and reproduction, increasing photorespiration and transpiration rates, altering pollen viability and fertilization, and disrupting metabolic processes (Zhang *et al.*, 2022). Plants are particularly sensitive to high temperatures during their reproductive stages, which can influence gene expression at the chromatin level and the circadian clock (Chang *et al.*, 2014; Mody *et al.*, 2020), and alter their profiling in plant roots (Martins *et al.*, 2017). Moreover, it has been reported that transcriptional factors, including heat shock proteins, are key in regulating gene expression networks involved in plant heat stress responses (Li *et al.*, 2019; Tolosa and Zhengbin, 2020; Pardo-Hernández *et al.*, 2024), as well as second messengers during signal transduction, protein folding, and protein–protein interactions (Zhang *et al.*, 2022). Conversely, suboptimal temperatures can reduce enzymatic activity and biochemical reactions, thereby adversely affecting plant growth and development (Hasdai *et al.*, 2006). Furthermore, temperature can modulate plant defense responses through specific proteins, such as NB-LRR proteins (Zhu *et al.*, 2010). In addition to plant growth and development, rising temperatures can indirectly influence crop yield by altering plant disease progression and insect pest biology (Jones, 2016; Jeger *et al.*, 2018; Jones and Naidu, 2019; Trebicki, 2020; Tonnang *et al.*, 2022). Accumulating evidence suggests that crops are vulnerable to viral diseases at high temperatures (Tsai *et al.*, 2022a, b; Iqbal *et al.*, 2023). In this sense, it has been reported that temperature can influence plant–virus interactions, affecting symptom expression and virus accumulation during the plant infection process (Aguilar *et al.*, 2015; Obrępalska-Stęplowska *et al.*, 2015; Chung *et al.*, 2016). For instance, studies have suggested that virus accumulation is temperature dependent, with seasonality affecting virus–plant interactions and virus dynamics during persistent infections (Honjo *et al.*, 2020), thus shaping viral genetic diversity and population dynamics in mixed infections (Alcaide *et al.*, 2021; Sardanyés *et al.*, 2022). Other studies have shown that exposure to elevated temperatures may either enhance or reduce plant susceptibility to viral diseases (Prasch and Sonnewald, 2013; Ghandi *et al.*, 2016; Makarova *et al.*, 2018; Amari *et al.*, 2021; Tsai *et al.*, 2022a). However, temperature can also affect the distribution of vectors that transmit plant viruses, leading to rapid and widespread dissemination (Jones, 2016; Islam *et al.*, 2020; Trebicki, 2020). Hence, rising temperatures could accelerate epidemic development and hinder disease management (Jones and Naidu, 2019). However, despite evidence that temperature affects several crops (Bita and Geräts, 2013; Grossiord *et al.*, 2020; Karki *et al.*, 2021; Rangaswamy *et al.*, 2021; Nguyen *et al.*, 2024) and that viral diseases may also elicit specific responses in plants, their combined effects on gene expression related to developmental and defense mechanisms remain unclear, as they have been extensively studied individually.

**Fig. 1.**
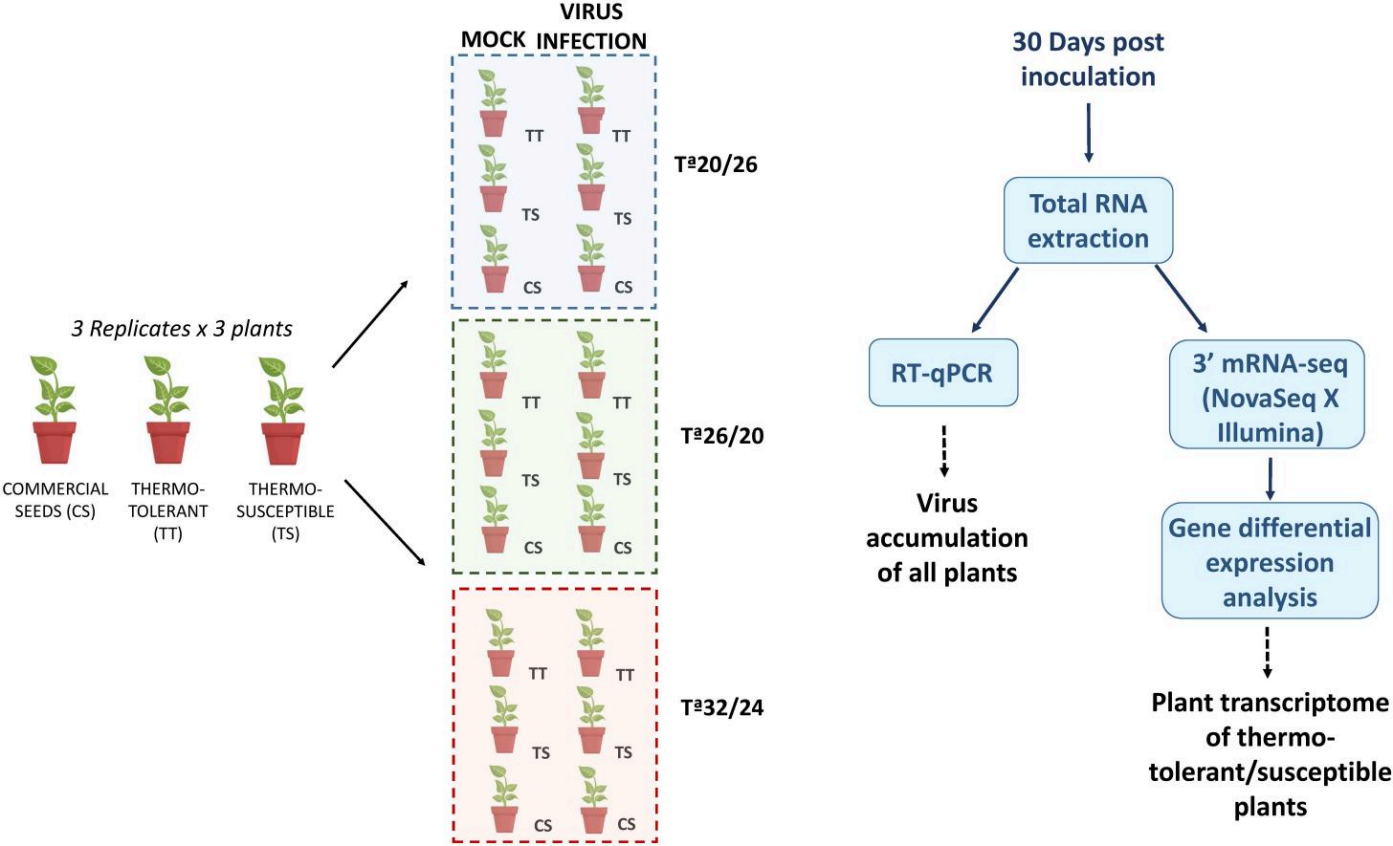
Schematic representation of the experimental design and workflow using thermotolerant/susceptible melon and zucchini plants. Three replicates, each consisting of a pool of three plants, were maintained at low (20/14 °C), medium (26/20 °C), and high (32/26 °C) temperature ranges under both uninfected and WMV-infected conditions. Plant material was collected at 30 dpi, and WMV accumulation was determined using absolute quantitative RT-PCR. Additionally, differential gene expression analysis was conducted using 3′ mRNA-seq via Illumina (NovaSeq X).

To elucidate the interactive molecular responses to combined temperature stress and viral infection, this study examined the gene expression profiles of two important cucurbit crops (melon and zucchini) at high, medium, and low temperatures, along with the presence or absence of the same viral infection, *Potyvirus citrulli* (watermelon mosaic virus, WMV). Functional genomic studies of plant crops are challenging because of the lack of full genome information. Thus, only a few studies have reported specific genes involved in the regulation of the drought stress response in melon plants (Xing *et al.*, 2020; Yang *et al.*, 2020), and studies on genes involved in heat stress in cucurbit plants are limited. Understanding how temperature modulates gene expression in plants can allow the identification of genes and alleles that are useful for marker-assisted selection, which can help plant breeding programs to enhance crop resilience (Hill and Li, 2022; Shen *et al.*, 2022). However, the effects of these improvements on the development of heat-tolerant varieties may be largely contingent on different agroecosystems and pathogen infections (Savary *et al.*, 2019). In this context, 28 viruses have been reported to significantly affect cucurbit crops in the Mediterranean Basin (Lecoq and Desbiez, 2012; Radouane *et al.*, 2021). Among these, aphid-transmitted viruses are widely distributed and are particularly detrimental to crop production sustainability (Lecoq and Katis, 2014; de Moya-Ruiz *et al.*, 2023; Rabadán *et al.*, 2023). WMV is a vector-borne virus primarily transmitted by several aphid species that causes serious diseases in major cucurbit production areas globally (de Moya-Ruiz *et al.*, 2021, 2023; Rabadán and Gómez, 2023; Rabadán *et al.*, 2023). In this study, we examine two interconnected aspects of plant–virus interactions, viral load and host transcriptional response, with their potential implications. In particular, we comprehensively integrated and analyzed the viral accumulation and transcriptomic datasets of melon and zucchini plants with high- and low-temperature tolerance, subjected to three temperature ranges (low, medium, and high) and WMV infection (Fig. 1). After analyzing viral RNA accumulation, we conducted comparative transcriptomic (3′ mRNA-seq) analysis of single and combined stress responses, characterizing gene clusters with distinct transcription patterns associated with abiotic (temperature) and biotic (viral infection) stress. Additionally, we linked the gene profiles of key biological processes to the temperature- and virus-responsive pathways in melon and zucchini plants, which could help develop effective strategies to improve cucurbit productivity.

## Materials and methods

### Plant material and growth conditions

Our experimental approach combines viral accumulation and transcriptomic analysis to establish causal relationships between two cucurbit plant species and virus infection responses to thermotolerance. This study utilized nine plants each of the thermotolerant (TT) and thermosusceptible (TS) melon and zucchini varieties (three replicates×three plants). Due to confidentiality agreements and potential conflicts of interest, the specific seed details provided by the respective plant breeding companies cannot be disclosed. Additionally, 54 commercial plants [nine plants each of melon (Piel de sapo) and zucchini (Black Beauty) varieties] were included and grown under the same conditions. Cucurbit seeds were initially sown in Petri dishes at 28 °C in darkness, and after 2 d were transplanted into 1 liter plastic pots filled with the substrate (coconut fiber, peat, and perlite in a 5:10:1 ratio). Each experiment was carried out independently in a controlled greenhouse with a 16/8 h photoperiod (16 h light and 8 h dark) at the respective experimental temperatures: 20 °C/16 °C (low), 26 °C/20 °C (medium), or 32 °C/24 °C (high), with ±6 °C of variation between experiment and day and night conditions. Despite the temperature and light regulations, note that the 26 °C experiment was conducted from April to June, the 32 °C experiment was conducted from July to September, and the 20 °C experiment was conducted from October to December, aligning with the natural seasonal temperatures in the region of Murcia, Spain. As a control, mock-treated plants were similarly subjected to temperature stress without viral infection. Based on our previous study on WMV accumulation in cucurbit plant species (de Moya-Ruiz *et al.*, 2021), the material was collected at 30 days post-infection (dpi) as the optimal time for viral performance and further analysis (Fig. 1).

### WMV virion purification and inoculation

Plants were inoculated with WMV virion particles. Briefly, first *Nicotiana benthamiana* plants were agroinoculated with WMV-MeWM7 (de Moya-Ruiz *et al.*, 2021) and maintained in a growth chamber at 24 °C. After 20 dpi, ∼60 g of symptomatic leaves were collected and ground in extraction buffer with liquid nitrogen, following a series of centrifugations and polyethylene glycol (PEG) precipitation, as described by Rupar *et al.* (2013), with some modifications. First, the homogenate was mixed with 5.38 ml g^–1^ of homogenization solution (0.5 M K_2_HPO_4_, 5 mM EDTA, 10 mM DIECA, and Na_2_SO_3_) and agitated at 4 °C for 15 min. The mixture was then centrifuged at 8500 *g* for 10 min at 4 °C. The supernatant was collected and filtered through gauze, Triton X-100 was added to a final concentration of 1%, and the mixture was shaken at 4 °C for 1 h. The supernatant was ultracentrifuged at 70 000 *g* for 90 min at 4 °C. After discarding the supernatant, the pellet was resuspended in 14 ml of citrate solution pH 7.5 +1% Triton X-100, and shaken for 30 min at 4 °C. The pellet was resuspended in 6 ml of citrate solution pH 7.5 +1% Triton X-100, shaken for 30 min at 4 °C, and centrifuged at 8500 *g* for 10 min. The supernatant was collected, and 10% chloroform was added, mixed thoroughly, and centrifuged at 8500 *g* at 4 °C for 10 min. The aqueous phase was collected and ultracentrifuged using a 30% sucrose cushion at 70 000 *g* for 2 h at 4 °C. The pellet was resuspended in 1 ml of citrate solution and incubated overnight at 4 °C. The purified virus was stored at −20 °C. For plant inoculation with WMV, the third and fourth true leaves were rubbed with carborundum powder and a suspension of 100 mg ml^–1^ virion particles in sodium phosphate buffer (30 mM, pH 8) (Gomez *et al*., 2009). The material (all apical leaves from each plant) was collected at 30 dpi from nine plants (three replicates×three plants) per treatment.

### Quantification of viral RNA accumulation

To determine WMV accumulation, samples were collected 30 dpi. Total RNA was extracted from all samples using Tri-reagent, purified by phenol–chloroform extraction, and treated with DNase I (Sigma-Aldrich, St. Louis, MO, USA). Viral accumulation was then quantified by absolute real-time quantitative PCR (RT-qPCR) with an AB7500 System (Applied Biosystems, Foster City, CA, USA) using the One-step NZYSpeedy RT-qPCR Green kit, ROX plus (NZYTech, Lisboa, Portugal) and two specific primers targeting the WMV P1 region (Supplementary Table S1). Briefly, the reaction mixture was performed in a total volume of 10 µl, containing 5 µl of One-step NZYSpeedy qPCR Green master mix (2×), ROX plus, 0.4 µl of NZYRT mix, 0.4 µl of reverse and forward primers, 1.8 µl of sterile water, and 2 µl of RNA. The PCR cycling protocol consisted of 50 °C for 20 min, 95 °C for 5 min, followed by 40 cycles of 95 °C for 5 s, 30 s at 60 °C annealing temperature, and the melting curve. Non-template controls were included to ensure product-specific amplification and the absence of primer dimers. Serial dilutions (10-fold) of viral RNAs from WMV-MeWM7 infectious clones were used to generate external standard curves (de Moya-Ruiz *et al.*, 2021). The initial RNA concentration was measured twice using a Qubit 3.0 fluorometer following the manufacturer’s instructions (Thermo Fisher Scientific). The RNA concentration in each sample (ng of viral RNA per 100 ng of total RNA) was estimated by plotting the threshold cycle (CT) values from each biological assay (*n*=9 at each time point) with three experimental replicates for each biological replicate. Given that the viral load of WMV in melon and zucchini plants had a similar exponential pattern, consistent with our previous studies (de Moya-Ruiz *et al*., 2021), and the most significant differences between temperatures were observed after 30 dpi, subsequent analyses focused on samples from this time point to ensure the reliability of the results and capture the relevant biological effects of each temperature condition.

### 3′ mRNA-sequencing and comparative analysis of gene expression profiles

Total RNA was extracted from samples of melon and zucchini at 30 dpi using Tri-reagent, purified by phenol–chloroform extraction, and treated with DNase I (Sigma-Aldrich). The quantity and quality of the RNA were assessed using a NanoDrop ND-1000 spectrophotometer (Thermo Fischer Scientific, Waltham, MA, USA) and agarose gel electrophoresis. The 3′ mRNA-sequencing was performed using NovaSeq X (Illumina Platform) by Seqplexing (Paterna, Valencia). This RNA-seq approach involves tagging the 3′ end of mRNA poly(A) tails with universal adapters, barcodes, and unique molecular identifiers, allowing for the accurate quantification of gene expression profiling (Charpentier *et al.*, 2021). Briefly, the bioinformatics pipeline starts by cleaning the raw sequence data by quality trimming and removing sequencing adapters and poly(A) sequences. Quality assessment was conducted using FastQC to ensure high-quality FASTQ files. UMIs were identified using umi-tools, duplicates were removed, and reads were mapped to the reference genome using STAR. The version of the genomic reference for zucchini and melon used were ‘Cpepo_genome_v4.1’, with the associated transcript annotation being ‘Cpepo_4.1’, both files obtained from http://cucurbitgenomics.org, and ‘Harukei3_v1.41’ with the associated transcript annotation being ‘Harukei3_v1.41’, both files obtained from https://melonet-db.dna.affrc.go.jp/ap/top, respectively.

HTSeq-count and counts were normalized to the total number of identified reads in each sample. This inter-sample normalization was followed by control sample normalization, including control samples (i.e. mock-treated plants under the same stress conditions) for each dataset, to enable comprehensive comparisons of the gene expression profiles of the thermotolerant and thermosusceptible melon and zucchini cultivars. The comparisons, along with the number of differentially expressed genes (DEGs) and total genes from both melon and zucchini cultivars, are summarized in Table 1. DESeq2 was employed to identify significant expression differences, identifying genes with an adjusted *P*-value<0.05, and log_2_fold change (FC) < −1 or >1 for each variety and crop. The variability between groups was analyzed using principal component analysis (PCA) conducted using iDEP 2.0 (http://bioinformatics.sdstate.edu/idep/) (Ge *et al.*, 2018). Upset plots and Venn diagrams were created using ChiPlot (https://www.chiplot.online/) and the R package, respectively.

**Table 1.** Summary of the 3′ mRNA-seq experiment design and results of DEGs for each condition including up- and down-regulated genes under the *P*-value <0.05 and log_2_FC<−1 or >1 criteria in melon and zucchini cultivars

Crop	Comparison	Total DEGs/total genes	%DEGs	DEGs (*P*-value<0.05/log_2_FC < −1 or >1)	
				Up-regulated	Down-regulated
*Cucumis melo*	TT Melon MOCK Ta20 versus TT Melon WMV-Ta20	14 955/37 254	40.14	337	119
	TT Melon MOCK Ta26 versus TT Melon WMV-Ta26	14 882/37 254	39.94	115	220
	TT Melon MOCK Ta32 versus TT Melon WMV-Ta32	15 351/37 254	41.20	519	1102
	TS Melon MOCK Ta20 versus TS Melon WMV-Ta20	15 150/37 254	40.66	118	110
	TS Melon MOCK Ta26 versus TS Melon WMV-Ta26	10 643/37 253	28.56	50	115
	TS Melon MOCK Ta32 versus TS Melon WMV-Ta32	10 544/37 253	28.30	133	280
	TT Melon MOCK Ta20 versus TT Melon MOCK Ta26	15 819/15 819	42.46	763	682
	TT Melon MOCK Ta32 versus TT Melon MOCK Ta26	15 299/15 299	41.06	758	477
	TS Melon MOCK Ta20 versus TS Melon MOCK Ta26	15 478/15 478	41.54	1126	897
	TS Melon MOCK Ta32 versus TS Melon MOCK Ta26	15 029/15 029	40.34	820	390
*Cucurbita pepo*	TS Zucchini MOCK Ta20 versus TS Zucchini WMV Ta20	21 938/27 868	78.72	109	299
	TS Zucchini MOCK Ta26 versus TS Zucchini WMV Ta26	15 963/27 868	58.28	183	295
	TS Zucchini MOCK Ta32 versus TS Zucchini WMV Ta32	16 281/27 868	58.42	366	640
	TT Zucchini MOCK Ta20 versus TT Zucchini WMV Ta20	10 825/27 868	38.84	227	119
	TT Zucchini MOCK Ta26 versus TT Zucchini WMV Ta26	17 169/27 868	61.60	279	256
	TT Zucchini MOCK Ta32 versus TT Zucchini WMV Ta32	16 332/27 868	58.60	101	497
	TS Zucchini MOCK Ta20 versus TS Zucchini MOCK Ta26	16 722/16 722	60.00	1772	2741
	TS Zucchini MOCK Ta32 versus TS Zucchini MOCK Ta26	16 101/16 101	57.77	212	108
	TT Zucchini MOCK Ta20 versus TT Zucchini MOCK Ta26	15 864/15 864	56.92	2827	3337
	TT Zucchini MOCK Ta32 versus TT Zucchini MOCK Ta26	15 061/15 061	50.04	652	975

### Functional annotation and Gene Ontology term enrichment analysis

Functional annotation of specific DEGs found in each melon and zucchini variety under a combination of temperature and virus infection was extracted from AmiGO 2 (https://amigo.geneontology.org/amigo/landing). Using the taxonomy ID 3656 and 3664 for melon and zucchini, respectively, the visualization platform of iDEP 2.0 (http://bioinformatics.sdstate.edu/idep/) and ShinyGO 0.77 (http://bioinformatics.sdstate.edu/go/) were used to perform GO enrichment analysis and produce a network of GO terms, using all available gene sets with a false discovery rate (FDR) cut-off of 0.05, and log_2_FC < −1 or >1. The top 15 pathways were considered.

### Identification and validation of orthologous genes

To identify orthologous genes between melon and zucchini under different temperature conditions and viral infections, reference genomes were obtained from the public Cucurbit Genomics Database (CuGenDB) (http://cucurbitgenomics.org/) (Yu *et al.*, 2023). We selected the well-annotated reference genomes of melon (DHL92) v3.6.1, and *Cucurbita pepo* subsp. Pepo. Orthologous genes were identified using the genes involved in the top 15 pathways using the Synteny Viewer tool. Furthermore, RT-qPCR was carried out to determine the expression of both orthologous genes by using an AB 7500 System (Applied Biosystems, CA, USA), and the One-step NZYSpeedy RT-qPCR Green kit, ROX plus (NZYTech, Lisboa, Portugal) as described above. Three biological replicates (each consisting of a pool of three plants) per treatment and two technical replicates were included for RT-qPCR analysis. According to the extensive evaluation of 14 and 13 candidate reference genes performed by Kong *et al*. (2014) in melon and Obrero *et al*. (2011) in zucchini, we selected the gene α-tubulin, which showed *M* values among the most stable genes under abiotic and biotic stress conditions. Thus, the primers CmTUA (Kong *et al.*, 2014) and TUA (Obrero *et al.*, 2011) were used for melon and zucchini plants, respectively (Supplementary Table S1). Note that we also observed consistent *C*_q_ values for TUA across all treatment conditions, with minimal variation between samples, confirming its suitability as a reference gene for our particular experimental setup. Primers to quantify melon and zucchini transcripts were designed using Primer3 software from the corresponding sequences available in the cucurbit genomics database (http://cucurbitgenomics.org). The primers used for MELO3C023308.2, MELO3C024920.2, Cp4.1LG05g12560, and Cp4.1LG06g08450 amplification are described in Supplementary Table S1. The fold change was calculated using the 2−ΔΔC_t_ method, with *T* 26, 20, and 32 °C, and without virus infection treatments used as the control under each viral condition, as described in Schmittgen and Livak (2008).

### Statistical analyses

Analysis of the viral load for each plant species was performed using two-way ANOVA. The viral accumulation data were transformed using a logarithmic function to achieve normality. The model included plant species (melon and zucchini) and temperature as two-level fixed effects. All analyses were performed using JMP software. Plot graphs of viral RNA accumulation for each isolate and plant species were generated using R software. Significant changes in transcript expression as compared with the control (DEGs) were defined as log_2_FC < −1 or >1, and adjusted *P*<0.05 (negative binomial Wald test followed by Benjamini–Hochberg correction) (Ferreira and Zwinderman, 2006). The variation in expression of the orthologous genes in melon and zucchini plants was analyzed using the Shapiro–Wilk normality test and the Student *t*-test (*P*<0.05), and plot graphs were generated using the ggplot2 package of R software.

## Results and discussion

### Watermelon mosaic virus load was significantly influenced by melon and zucchini plants and depended on their thermotolerance

**Fig. 2.**
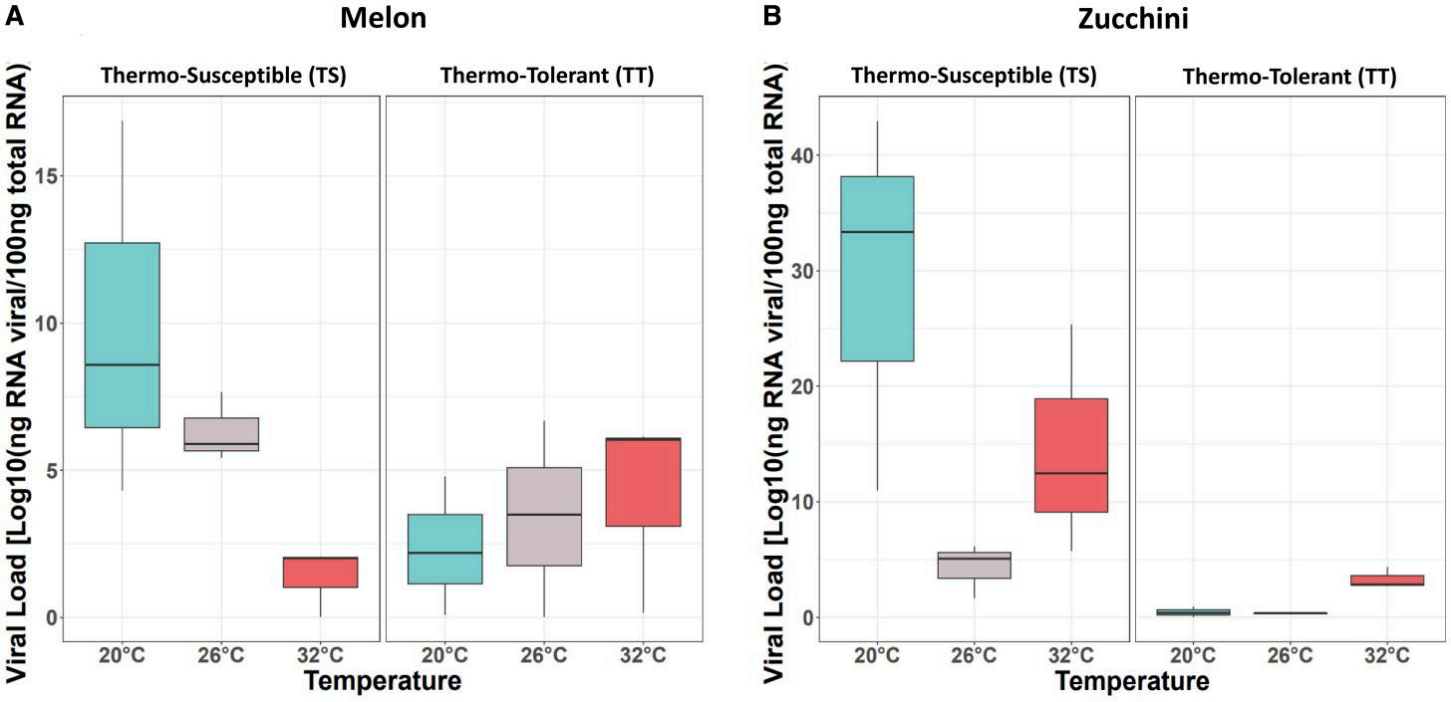
Viral load (mean and SE error bars, *n*=3) of WMV infectious clones (MeWMV7) in thermosusceptible (TS) and thermotolerant (TT) melon (A) and zucchini (B) plants at 30 dpi under three different growth temperatures: 20 °C/16 °C (low), 26 °C/20 °C (medium), or 32 °C/24 °C (high). Viral RNA accumulation was determined using RT-qPCR. RNA transcripts of P1 were serially diluted (10-fold) to generate external standard curves. The RNA concentration in each sample (ng of viral RNA per 100 ng of total RNA) was estimated from the cycle threshold (C_t_) values obtained from each independent biological assay, with three biological replicates at each time point.

To determine the extent to which temperature affects viral accumulation in melon and zucchini plants, the WMV load was estimated using absolute RT-qPCR in commercial melon (Piel de sapo) and zucchini (Black beauty) cultivars grown at different temperatures (low, 20/14 °C; medium, 26/20 °C; and high, 32/26 °C). At 30 dpi, WMV had 4-fold greater accumulation in zucchini than in melon plants (Supplementary Fig. S1A, B; *F*_1,12_=193.23, *P*<0.001), with a significant interaction between temperature and plant species (*F*_2,12_=78.09, *P*<0.001). This viral accumulation pattern may have arisen because of the host specificity and limited viral fitness of WMV in melon plants at high temperatures. Many studies have shown that viral titers can be influenced by temperature. For example, turnip mosaic virus (TuMV) and plum pox virus (PPV) show lower accumulation at high temperatures, with milder symptoms (Szittya *et al.*, 2003; Aguilar *et al.*, 2015; Chung *et al.*, 2015). In contrast, TuMV has been observed to exhibit more severe symptoms in Chinese cabbage at 28 °C, which correlates with higher viral accumulation (Chung *et al.*, 2015). However, plants may experience a slowdown in metabolic processes at lower temperatures, which could affect the efficiency of plant defense mechanisms and, in turn, may affect viral replication and accumulation (Garcia-Ruiz, 2018). Therefore, we aimed to evaluate the effects of plant thermotolerance on viral accumulation at varying temperatures. Specifically, we examined WMV accumulation in melon and zucchini plants with different levels of thermotolerance (TT, thermotolerant; and TS, thermosusceptible) at three temperature ranges (low, medium, and high). In melon plants, the WMV load decreased significantly in the TS cultivar as the temperature increased, with a 7-fold reduction in the high-temperature range (*F*_2,6_=6.12; *P*=0.003). In contrast, the WMV load in the TT melon cultivar did not vary significantly across temperature ranges (*F*_2,6_=0.11, *P*=0.89). However, in zucchini plants, the WMV load was generally higher than that in melons, and its accumulation pattern was reversed. In TS zucchini plants, the WMV load remained relatively similar across the three temperature ranges (*F*_2,6_=4.62; *P*=0.061), whereas in TT zucchini plants, it significantly increased in the high-temperature range (*F*_2,6_=19.90; *P*=0.002) [Fig. 2A, B (TS versus TT); two-way ANOVA]. These results indicate different responses to heat stress between the TS and TT cultivars in both cucurbit species, whereas WMV load showed a relative decrease with increasing temperature in TS plants, and TT plants exhibited certain resilience under temperature changes. These responses suggest that plant susceptibility to temperature affects viral accumulation. In this sense, it has been reported that RNA silencing-mediated defense can be inhibited at low temperatures (Szittya *et al.*, 2003; Chellappan *et al.*, 2005), which in turn can increase virus accumulation. However, temperature can also affect phytohormone-mediated defense pathways, influencing RNA silencing and virus-encoded silencing suppressors (Lewsey *et al.*, 2010). Other studies have shown that high temperatures may increase the susceptibility of tomato plants to tomato yellow leaf curl virus (TYLCV), Arabidopsis to TuMV, or potato to potato virus Y (PVY) (Prasch and Sonnewald, 2013; Ghandi *et al.*, 2016; Fesenko *et al.*, 2021). In the latter case, and in contrast to our results, these studies found that rising temperatures led to greater accumulation of PVY in TS than in TT plants, possibly due to the reduction of pathogenesis-related proteins [salicylic acid (SA)-mediated plant defense] (Makarova *et al.*, 2018; Spechenkova *et al.*, 2021). It is likely that an increase in temperature can reduce plant resistance, as in the case of tobacco mosaic virus (TMV) and potato potexvirus X (PVX), where an increase in temperature leads to a reduction in the induced hypersensitivity response (Wang *et al.*, 2009). However, it has also been reported that temperatures above 28 °C can induce a suppression of virus-induced hypersensitive response-type necrosis caused by TMV (Király *et al.*, 2008). In addition, increased temperature and faster symptom development could also be correlated with virus accumulation, as observed for peanut stunt virus (PSV) in *N. benthamiana* (Obrępalska-Stęplowska *et al.*, 2015), PVY in potato (Makarova *et al.*, 2018), and capsicum chlorosis virus in pepper (Tsai *et al.*, 2022a). Taken together, our results suggest that temperature can influence WMV accumulation in melon and zucchini plants based on their thermotolerance levels. This outcome could be related to differences in the expression of genes and proteins involved in stress responses, which could indirectly affect viral replication, movement, and/or accumulation within plants.

### Variations in the number and distribution of differentially expressed genes across temperature ranges and watermelon mosaic virus infections between melon and zucchini plants

To examine the effect of WMV infection and temperature variations (low, medium, and high) on the gene response of melon and zucchini with different temperature tolerances (TT and TS), we carried out a differential 3′ mRNA-seq approach of plants under each temperature condition in the presence or absence of WMV. The reads obtained from each replicate sample were normalized and analyzed by PCA. Overall, PCA clustered mock samples separately from virus-infected samples, as well as by temperature conditions within the TS and TT melon and zucchini samples (Supplementary Fig. S2A, B). After bioinformatic processing of the total DEGs, and the subsequent analysis using the criteria *P*<0.05 and log_2_FC ≤ −1 or≥1, we found a total of 14 955 DEGs at low (337 overexpressed and 119 underexpressed), 14 882 at medium (115 overexpressed and 220 underexpressed), and 15 351 at high (519 overexpressed and 1102 underexpressed) temperatures in TT melon, whereas 15 150 DEGs were found at low (118 overexpressed and 110 underexpressed), 10 643 at medium (50 overexpressed and 115 underexpressed), and 10 544 at high (133 overexpressed and 280 underexpressed) temperatures in TS melon, out of 37 254 annotated genes in melon. In zucchini plants, we found 21 938 DEGs (109 overexpressed and 299 underexpressed) at low, 15 963 at medium (183 overexpressed and 295 underexpressed), and 16 281 at high temperatures (366 overexpressed and 640 underexpressed) in TS zucchini, whereas 10 825 DEGs (227 overexpressed and 119 underexpressed) were found at low, 17 169 at medium (279 overexpressed and 256 underexpressed), and 16 332 at high temperatures (101 overexpressed and 497 underexpressed) in TT zucchini, out of 27 868 annotated genes (Supplementary Tables S2–S4 include additional details). Thus, zucchini plants had an ∼2.6-fold higher percentage of DEGs compared with melon (*F*Ratio=17.9602 and Prob>*F*=0.0028). In both TS and TT plants, the percentage of DEGs ranged from 10 825 (39%) to 21 938 (79%) out of 27 868 annotated genes in zucchini, and from 10 544 (28%) to 15 351 (41%) out of 37 254 annotated genes in melon (Table 1). The highest percentage of DEGs was exhibited in TS zucchini plants (79%) at lower temperatures. It is likely that this high percentage of DEGs in TS at low temperatures may be related to the greater accumulation of WMV, suggesting specific transcriptional responses of TS to the combination of temperature and virus infection. This appeared to be consistent with previous studies that have shown that low-temperature conditions make plants more susceptible to viruses because of the inhibition of RNA silencing-mediated defence by controlling siRNA generation (Szittya *et al.*, 2003; Tenllado and Canto, 2020).

**Fig. 3.**
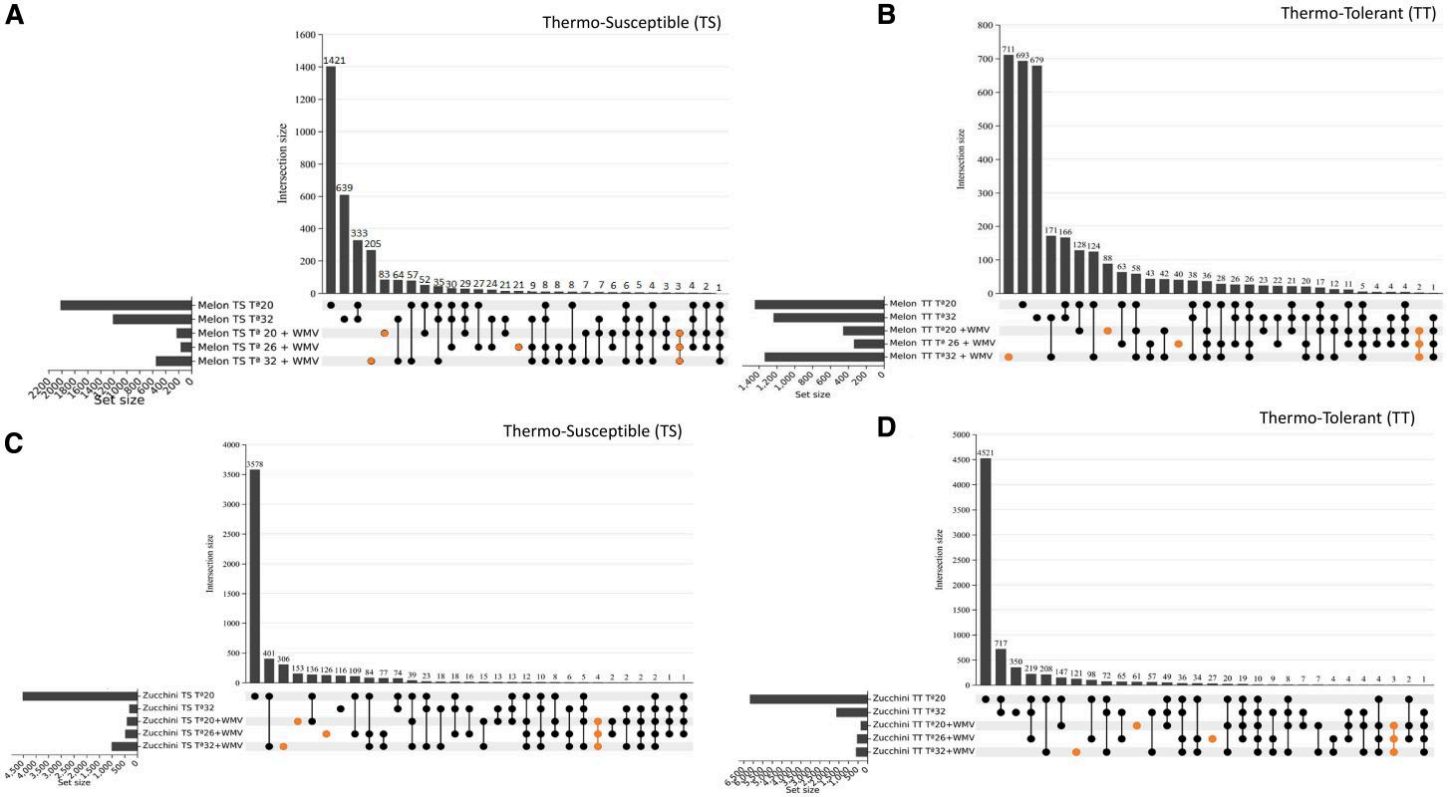
Upset plot displaying intersections between sets of DEGs found under the combined stress effect in thermosusceptible melon (A) and zucchini (C) plants, and in thermotolerant melon (B) and zucchini (D) plants. Up- and down-regulated genes for each plant species, thermotolerance, and stress condition were placed in the same set for subtraction of the specific DEGs under the combination of temperature and WMV infection. The bar plot at the top represents the overlap of DEGs under each condition. The horizontal bar on the left represents the number of significant DEGs under each condition. The black circles and lines at the bottom show the categories for which overlap was calculated, as indicated in the bar chart at the top. The orange circles highlight the DEGs exclusive to each condition in combination with both stresses (WMV+L, WMV+M, and WMV+H), and the overlap of significant DEGs observed in the three conditions. It should be noted that low- and high-temperature (20 °C and 32 °C) conditions were only considered as a reference to rule out the specific number of DEGs in the combination of low and high -temperature and WMV infection, while the medium temperature (26 °C) was included in combination with WMV infection to identify the common DEGs as a response to the WMV infection regardless of the three temperature conditions.

We then sought to identify unique genes under both viral infection and heat stress conditions. In both TS and TT melon and zucchini samples, the highest number of unique DEGs was observed under the conditions of WMV infection and high temperature (WMV+H) (Fig. 3A–D; Supplementary Table S2). Specifically, under WMV+H conditions, TT and TS melon plants exhibited 711 and 205 genes, respectively, whereas 40 and 21 genes were unique under WMV+M conditions, and 88 and 83 genes were unique under WMV+L conditions. In TT and TS zucchini plants, 121 and 306 genes were unique to the WMV+H condition, respectively, while, 27 and 126 genes were unique to WMV+M, and 61 and 153 genes to WMV+L, respectively (for more detailed information, see Supplementary Fig. S3). This indicated that the effect of WMV infection at high temperatures could be associated with a greater number of unique DEGs in TT melon plants and in TS zucchini plants, when compared with the medium and low temperature ranges. Note that only a few (2–4) DEGs were found to be common for the three temperature ranges and viral infection, suggesting that those genes might be attributed to the single WMV infection (Fig. 3A–D; Supplementary Table S2). Some plant viral infections have been suggested to mitigate the detrimental effects of abiotic stress (Gorovits *et al.*, 2019; Aguilar and Lozano-Duran, 2022; Mishra *et al.*, 2022). For example, TYLCV infection in tomato plants can suppress the heat shock response, alleviate the response of plant cells to heat stress, prevent cell death, and allow plants to adapt to high temperatures and water deficits (Corrales-Gutierrez *et al.*, 2020; Gorovits *et al.*, 2022). Alternatively, TuMV infection has been shown to reduce stomatal conductance, leading to altered expression levels of abscisic acid (ABA) homeostatic genes, including biosynthesis and catabolism, eventually improving drought tolerance in Arabidopsis (Manacorda *et al.*, 2021). Therefore, specific signaling pathways may be involved in these biotic and abiotic processes, and further research is needed to identify potential targets and fully understand this complex interplay.

### Changes in biological processes and molecular functions in response to WMV infection and temperature

**Fig. 4.**
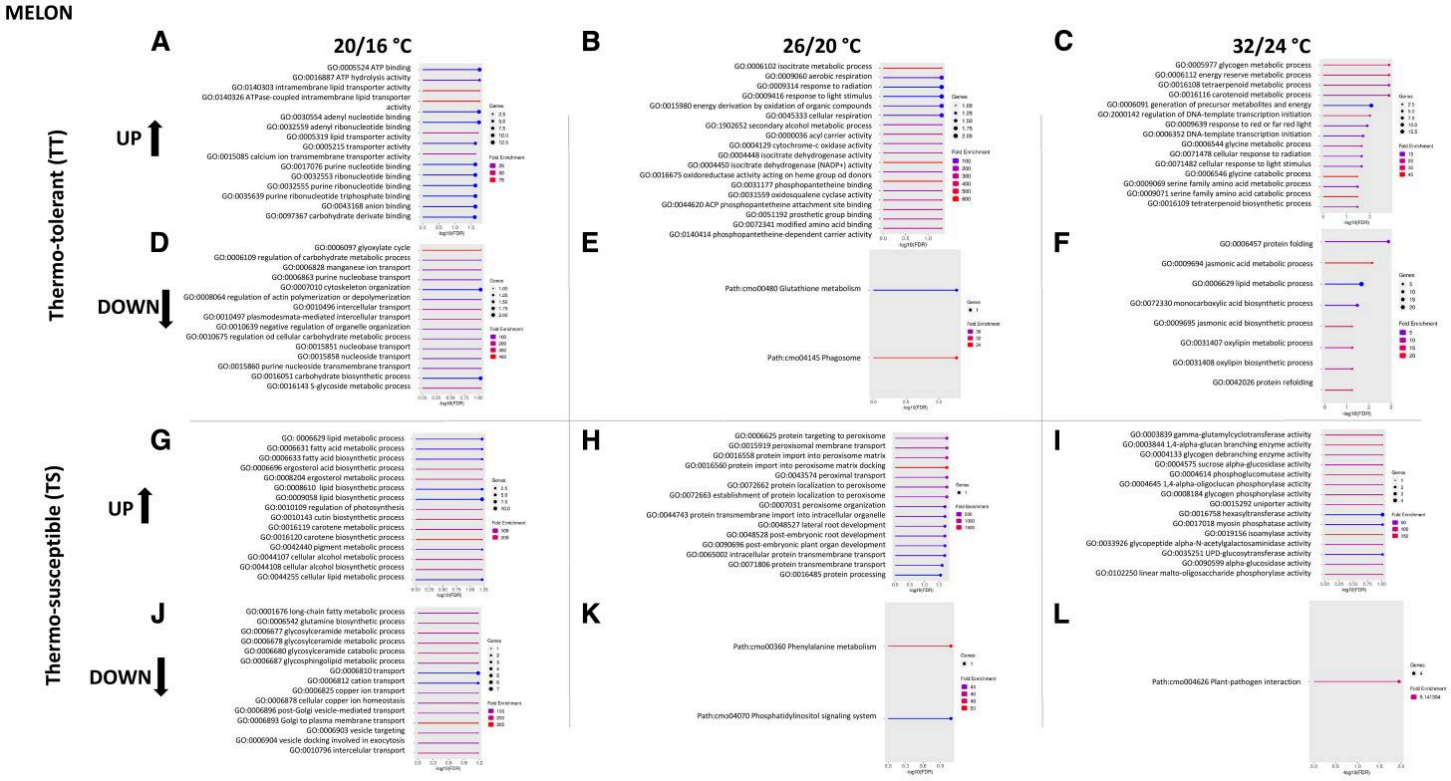
Gene Ontology (GO) enrichment analysis for the top 15 GO terms of specific DEGs (using the criteria *P*<0.05 and log_2_FC ≤ −1 or ≥1) in melon plant varieties under the combination of temperature+WMV infection based on biological process and molecular function. GO enrichment profiles are shown for thermotolerant up-regulated genes at low (A), medium (B), and high (C) temperatures, and down-regulated genes at low (D), medium (E), and high (F) temperatures. GO enrichment profiles are shown for thermosusceptible up-regulated genes at low (G), medium (H), and high (I) temperatures, and down-regulated genes at low (J), medium (K), and high (L) temperatures. All data are listed in Supplementary Table S7.

**Fig. 5.**
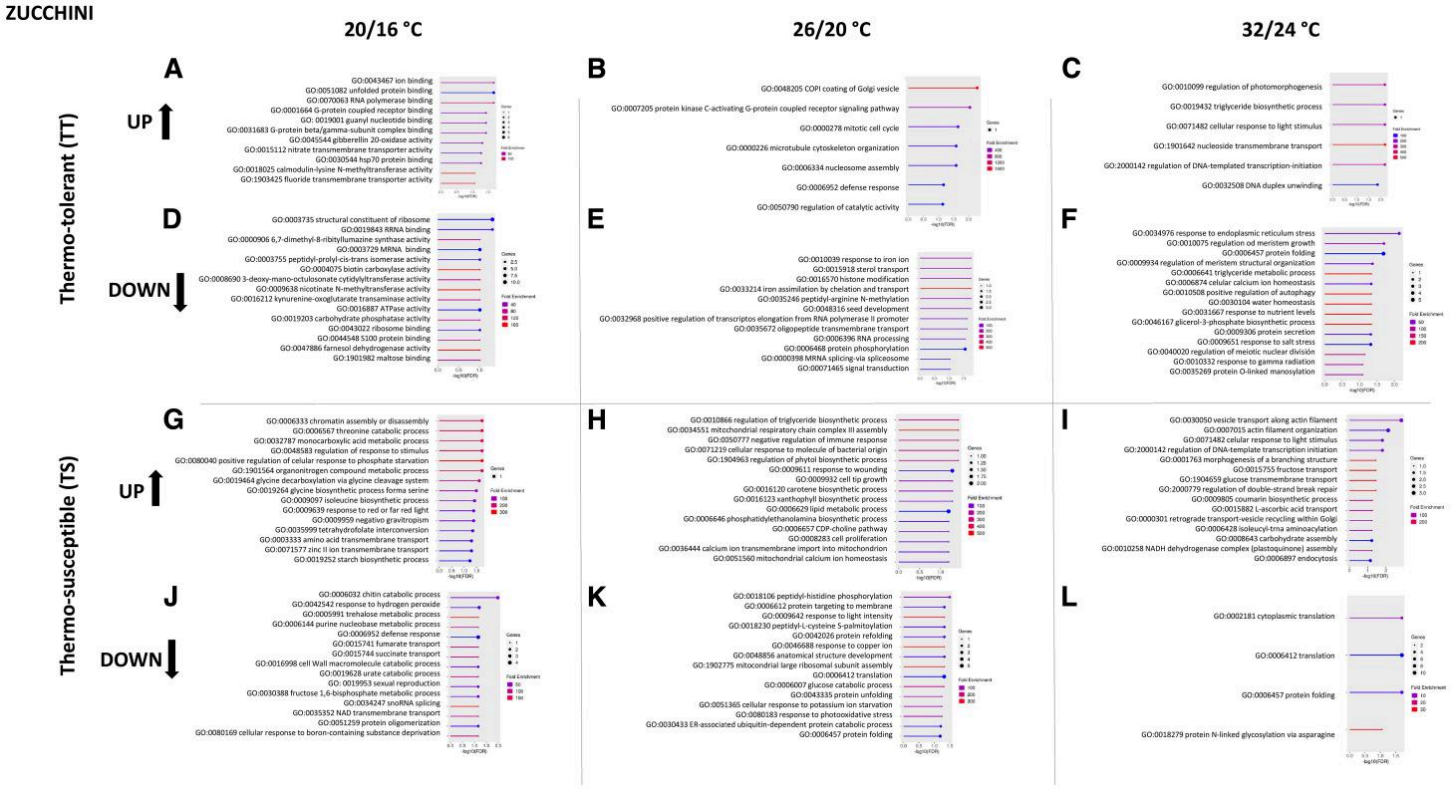
Gene Ontology (GO) enrichment analysis for the top 15 GO terms of specific DEGs (using the criteria *P*<0.05 and log_2_FC ≤ −1 or ≥1) in zucchini plant varieties under the combination of temperature+WMV infection based on biological process and molecular function. GO enrichment profiles are shown for thermotolerant up-regulated genes at low (A), medium (B), and high (C) temperatures, and down-regulated genes at low (D), medium (E), and high (F) temperatures. GO enrichment profiles are shown for thermosusceptible up-regulated genes at low (G), medium (H), and high (I) temperatures, and down-regulated genes at low (J), medium (K), and high (L) temperatures. All data are listed in Supplementary Table S8.

Having established the differential expression patterns, we next investigated how these host responses specifically influence viral infection and heat stress. Since functional genomic studies in cucurbits are ongoing and there are still transcripts that are poorly annotated in the Kyoto Encyclopedia of Genes and Genomes (KEGG) database, we used GO enrichment to assign DEGs with biological processes and molecular functions. GO analysis was performed for both up- and down-regulated sets of unique genes, which were selected according to the criteria of *P*<0.05, and log_2_FC ≤ −1 or ≥1, including an FDR adjustment of 0.05, as the threshold of significance. Among them (Supplementary Tables S3–S6), the top 15 pathways (up- and down-regulated) for each temperature condition were ranked by *P*-value (Fig. 4, melon; Fig. 5, zucchini), with more detailed information in Supplementary Tables S7 and S8. We found that a considerable percentage of DEGs were related to biotic and/or abiotic stress in all samples. In particular, in TT samples, 32, 23, and 48% of DEGs were found at low, medium, and high temperatures, respectively. Similarly, in the TS samples, 41%, 50%, and 41% were observed at low, medium, and high temperatures, respectively. This suggests that temperature had a greater impact on the DEGs in the TS samples. Note that the remaining enriched GO terms were related to other processes that were not currently associated with biotic and abiotic stress. Among the GO terms related to biotic stress, some annotated common DEGs were defence response (GO:0006952), Hsp70 protein binding (GO:0030544), plant–pathogen interaction (Path:cmo04626), and those related to jasmonic acid metabolism (GO:0009694 and GO:0009695) (Supplementary Tables S7 and S8). These particular genes have been reported to be involved in biotic stress responses (Jones and Dangl, 2006; Mittler *et al.*, 2012; Wasternack and Strnad, 2018). Similarly, among the GO terms related to temperature stress, the annotated DEGs were responses to radiation or light stimulus (GO:0009314 and GO:0009416) or cellular copper ion homeostasis (GO:0006878), and were reported to be involved in abiotic stress (Wang, 2005; Yruela, 2005; Hideg *et al.*, 2013). Furthermore, GO terms were related to both abiotic and biotic stressors, such as protein folding, secretion, processing (GO:0006457, GO:0009306, and GO:0016485), and lipid metabolism (GO:0006631) (Queitsch *et al.*, 2000; Narayanan *et al.*, 2016). Analysis of TT melon plants revealed that ATPase-coupled intramembrane lipid transporter activity (GO:0140326 and GO:0140303) and the glyoxylate cycle (GO:0006097) were enriched with up-regulated and down-regulated genes, respectively, in the low-temperature range (Fig. 4A, D). Specifically, in the case of ATPase-coupled intramembrane lipid transporter activity, GO-Term was strongly related to other highly significant factors, such as lipid transport (GO:0140303 and GO:0005319) (Supplementary Fig. S4A). In the medium-temperature range, the isocitrate metabolic process (GO:0006102) and phagosome (Path:cmo04145) were enriched with up-regulated and down-regulated genes, respectively (Fig. 4B, E), which are associated with and involved in similar biological processes and molecular functions (Supplementary Fig. S4B). While a large number of GO terms were enriched in the high-temperature range, highlighting the glycine catabolic process (GO:0006546) and serine family amino acid catabolic process (GO:0009071) with up-regulated genes (Fig. 4C), which are functionally related (Supplementary Fig. S4C), as well as jasmonic acid metabolic process (GO:0009694) with down-regulated genes (Fig. 4F) and related to jasmonic acid biosynthesis (GO:0009695) (Supplementary Fig. S4C). On the other hand, in TS melon, the carotene metabolic process (GO:0016120) and Golgi to plasma membrane transport (GO:0006893) were found to be up- and down-regulated genes, respectively, in the low-temperature range (Fig. 4G, J). The carotene metabolic process has been highly related to other GO terms, such as carotene biosynthetic process, and some of which are related to cellular alcohol (GO:0044107) and ergosterol process (GO:0008204 and GO:0006696) (Supplementary Fig. S4D). In the medium-temperature range, protein import into peroxisome docking (GO:0016560) and phenylalanine metabolism (Path:cmo00360) were enriched for up- and down-regulated genes, respectively (Fig. 4H, K) that are involved in similar biological processes and molecular functions (Supplementary Fig. S4E).

In the high-temperature range, isoamylase activity (GO:0019156), which has been associated with glycogen debranching enzyme activity (GO:0004133) (Supplementary Fig. S4F), and plant–pathogen interactions (Path:cmo04626) were found for up- and down-regulated genes, respectively (Fig. 4I, L).

**Fig. 6.**
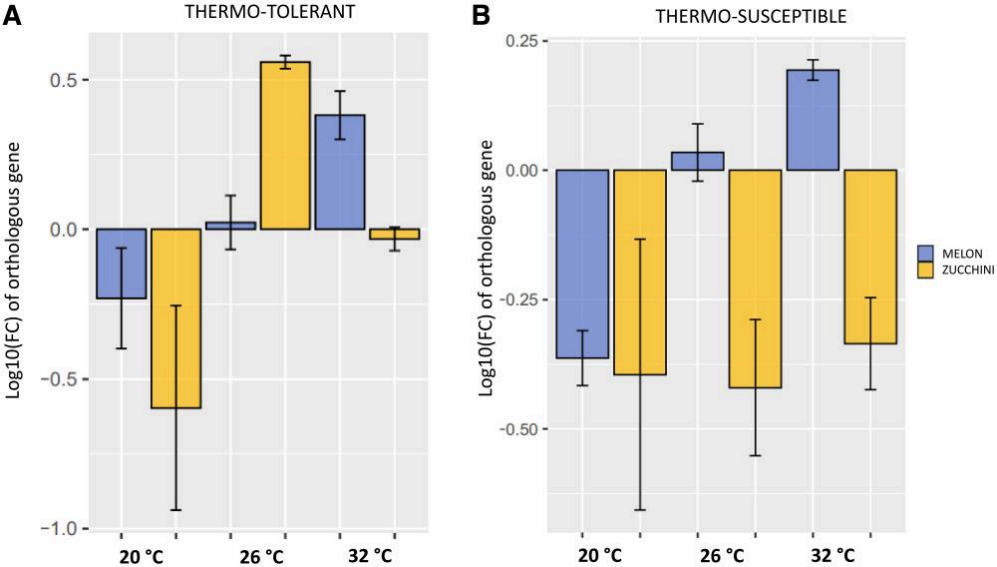
Relative expression of the unique melon and zucchini orthologous genes. Gene expression is represented as log10(FC), which was calculated using the 2−ΔΔC_t_ method for each temperature (low, medium, and high) in the WMV infection condition, and referenced to their corresponding mock conditions. A log10(FC) value of 0 indicated no change in gene expression, values >0 indicated an increase in expression, and values <0 indicated a decrease in expression. (A) Orthologous genes that were identified in TT melon (MELO3C023308.2, blue bars) and zucchini (Cp4.1LG05g12560, yellow bars) under high-temperature conditions. (B) Orthologous genes that were identified in TS melon (MELO3C024920.2, blue bars) and zucchini (Cp4.1LG06g08450, yellow bars) under low temperature. Bars represent the mean ±SD of three independent biological experiments, and statistical significance was assessed using the Shapiro–Wilk normality test and Student’s *t*-test (*P*<0.05).

Analysis of TT zucchini plants showed that calmodulin-lysine *N*-methyltransferase activity (GO:0018025) and fluoride transmembrane transporter activity (GO:1903425) were enriched in the up-regulated genes (Fig. 5A), and farnesol dehydrogenase activity (GO:0047886), nicotinate *N*-methyltransferase activity (GO:0008938), and biotin carboxylase activity (GO:0004075) were enriched among the down-regulated genes in the low-temperature range (Fig. 5D), none of which was associated with any of the top 15 pathways (Supplementary Fig. S5A). In the medium-temperature range, the COPI coating of Golgi vesicles (GO:0048205) and iron assimilation by chelation and transport (GO:0033214) were enriched in the up- and down-regulated genes, respectively (Fig. 5B, E), which are involved in similar biological processes and molecular functions (Supplementary Fig. S5B). In the high-temperature range, nucleoside transmembrane transport (GO:1901642), which is associated with nucleoside transmembrane transporter activity (GO:0005337) (Supplementary Fig. S5C), and triglyceride metabolic process (GO:0006641), which has been associated with the glycerol-phosphate biosynthesis process (GO:0046167) (Supplementary Fig. S5C), were enriched for up- and down-regulated genes, respectively (Figs 5C, 6F). In TS zucchini plants, the analysis showed positive regulation of the cellular response to phosphate starvation (GO:0080040) and trehalose metabolic process (GO:0005991), which have been associated with alpha-trehalase activity (GO:0004555) (Supplementary Fig. S5D), were enriched with up- and down-regulated genes, respectively, in the low-temperature range (Fig. 5G, J). In the medium-temperature range, mitochondrial respiratory chain complex III assembly (GO:0034551) and response to light intensity (GO:0009642), copper ion (GO:0046688), and mitochondrial ribosomal subunit assembly (GO:1902775) were enriched with up- and down-regulated genes, respectively (Fig. 5H, K) that are involved in similar biological processes and molecular functions (Supplementary Fig. S5E). In the high-temperature range, fructose and glucose transport (GO:0015755, GO:0005353, and GO:1904659), which have been strongly associated with similar biological processes and molecular functions (Supplementary Fig. S5F), and protein *N*-linked glycosylation via asparagine (GO:0018279) were also found to be enriched for up- and down-regulated genes, respectively (Fig. 5I, L). The identification of these genes suggests that certain biological processes may be linked to both abiotic and biotic stressors. Although it should be noted that our results could be influenced by the specific temperature range and pathogen used in our experimental design, it is evident that a combination of these stress factors can play an important role in plant physiology. It is also worth mentioning that despite the cucurbit genomics database (CuGenDB) being a pivotal and valuable resource for advancing comparative and functional genomic studies (Zheng et al., 2019), functional genomic studies in non-model crops are usually challenging, and further research is needed to elucidate the specific genes underlying abiotic and biotic stresses.

### Identification of unique orthologous genes in thermosusceptible and thermotolerant melon and zucchini plants infected with watermelon mosaic virus

To uncover genes uniquely responsive to combined temperature and virus stress, we first filtered orthologs among the top 15 GO terms for temperature and WMV infection (details listed in Supplementary Tables S7 and S8). Under the high-temperature range, two orthologous genes were found in thermotolerant plants, with significant transcriptomic changes: MELO3C023308.2 in melon (log_2_FC=1.709) and Cp4.1LG05g12560 in zucchini (log_2_FC)=1.036). To validate these transcriptomic results, we performed RT-qPCR for both orthologous genes using the gene α-tubulin as the endogenous control and compared relative expression across the temperatures and viral condition treatments (Fig. 6A). Statistical analysis confirmed a significant temperature by WMV infection interaction (interaction between temperature and TT plant species: *F*_2,2_=5.326, *P*=0.022). In particular, both orthologous genes were down-regulated at low temperature under WMV infection, while there was a significant up-regulation of MELO3C023308.2 at high temperature [*t*_(2)_=4.70 *P*=0.041], and also of Cp4.1LG05g12560 at medium temperature [*t*_(2)_=25.43, *P*=0.0015], with no significant changes at high temperature compared with its control (*P*=0.49). This confirmed the transcriptomic changes and suggested that these orthologous genes exhibit a temperature-dependent response under WMV infection. These genes encode F-box proteins, known to play pivotal roles in regulating the expression of genes involved in plant defense responses by recognizing and reacting with pathogen-associated molecular patterns (PAMPs) and effector-triggered immunity (ETI). They are involved in the regulation of protein stability during immune responses, ensuring efficient defense against pathogens, as well as in the degradation of misfolded or damaged proteins accumulated during temperature stress, thus maintaining cellular homeostasis and regulatory proteins that modulate heat stress and hypersensitive resistance responses (Xiao and Jang, 2000). This melon gene (MELO3C023308.2) is annotated with the following GO terms: GO:0009658, GO:0048512, GO:0045893, GO:0080167, GO:0010099, and GO:0009639, which are related to chloroplast organization, circadian behavior, positive regulation of transcription, response to karrikin, regulation of photomorphogenesis under the biological process category, and response to red or far red light, respectively. In the GO terms network, it was directly related to the regulation of DNA-templated transcription initiation (GO:2000142), cellular response to radiation (GO:0071478), and light stimulus (GO:0071482) (Supplementary Fig. S4C). In zucchini, the gene mentioned (Cp4.1LG05g12560) is annotated under the term GO:0010099, which is related to the regulation of photomorphogenesis under the biological process category. This is consistent with previous studies on *Arabidopsis thaliana*, which showed that thermomorphogenetic effects occur in response to high temperatures (Nomoto *et al.*, 2012; Yamashino *et al.*, 2013). These thermomorphogenetic responses are typical of TT varieties, making them attractive for crop breeders aiming to develop plants that can withstand climate change (Quint *et al.,* 2016).

Additionally, under the low-temperature range, another two orthologous genes were found in thermosuceptible plants: MELO3C024920.2 in melon (log_2_FC= −1.010) and Cp4.1LG06g08450 in zucchini (log_2_FC=1.258). Similarly, gene expression was also validated by RT-qPCR and under WMV infection. Both genes responded differentially (*F*_2,2_=2.152, *P*=0.158), with only MELO3C024920.2 affected by the temperature conditions (*F*_1,1_=10.313, *P*=0.075). In particular, MELO3C024920.2 was down-regulated at low temperature [*t*_(2)_= −6.82, *P*=0.020], while there was a significant up-regulation at high temperature [*t*_(2)_ = 9.84, *P*=0.01]. However, Cp4.1LG06g08450 remained significantly down-regulated at medium and high temperature (*P*<0.01) (Fig. 6B). This suggested that MELO3C024920.2 had a response dependent on temperature under WMV infection, while Cp4.1LG06g08450 exhibited a significant down-regulation regardless of the temperature, and possibly attributed to the WMV infection. These genes encode zinc transporters, which have been shown to be involved in the regulation of various signaling pathways that are crucial for plant growth and development, making them key players in plant stress responses. Zinc transporters regulate zinc uptake, transport, and distribution, influencing the ability to tolerate and adapt to adverse environmental conditions, such as drought, salinity, and heavy metal stress (Ullah et al., 2019), and the immune response of the plant against pathogens by regulating zinc accumulation (Cabot et al., 2019). The MELO3C024920.2 gene is annotated with the following GO terms: GO:0030001, GO:0055085, GO:0055114, GO:0071577, GO:0006810, and GO:0006812, which are related to metal ion transport, transmembrane transport, oxidation–reduction process, and zinc II ion transmembrane transport under the biological process category, transport, and cation transport, respectively. The GO term network was directly related to cellular copper ion homeostasis (GO:0006878) and transport (GO:0006825) (Supplementary Fig. S4D). In zucchini, the gene mentioned (Cp4.1LG06g08450) is annotated under the terms GO:0030001, GO:0055085, GO:0055114, and GO:0071577, which are related to metal ion transport, transmembrane transport, and oxidation–reduction processes under the biological process category.

Our results suggest that these orthologous genes may be crucial for maintaining the balance between stress and growth in plants, and further research should be conducted to explore their specific functions in different plant species under combined stresses. Curiously, in the medium-temperature range, no orthologous genes were found in TT or TS between the plant species, suggesting that these genetic traits might be limited to specific temperature thresholds. While recognizing the importance of validating these potential orthologous gene candidates across all plant conditions, it is worth noting that this effort is ongoing and requires further research. Additionally, collaboration with breeding company holders of these melon and zucchini cultivars is essential, as this work could significantly contribute to developing cucurbit varieties with enhanced tolerance to WMV infection and temperature stress. This suggests that the plant response to viral infection is closely linked to temperature stress. Additionally, the identification of orthologous genes that differentiate TS varieties from TT varieties may provide potential molecular targets for breeding programs. These findings underscore the importance of understanding the molecular interplay between biotic and abiotic stressors in the development of resilient crop varieties to support sustainable agriculture and food security.

## Supplementary Material

eraf277_Supplementary_Data

## Data Availability

All RNA sequencing (3′ mRNA-seq) data that support the findings of this study have been deposited in ArrayExpress (EMBL-EBI), with the accession codes E-MTAB-14116 for melon and E-MTAB-14118 for zucchini plant species. The raw data, processed data, and metadata with detailed sample annotations and protocols of this 3′ mRNA study are provided.

## Supplementary data

The following supplementary data are available at *JXB*  online.

Fig. S1. Viral load of WMV infectious clones in commercial melon zucchini plants at 30 dpi under three different growth temperatures.

Fig. S2. Principal component analysis (PCA) of thermosusceptible and thermotolerant melon and zucchini plants.

Fig. S3. Venn diagrams of the overlap of up- and down-regulated genes between thermotolerant and thermosusceptible melon plants and zucchini plants under each stress treatment.

Fig. S4. Pathway analysis and GO term networks based on molecular function and biological processes of specific DEGs under the combination of temperature+WMV infection in melon plant varieties

Fig. S5. Pathway analysis and GO term networks based on molecular function and biological processes of specific DEGs under the combination of temperature+WMV infection in zucchini plant varieties.

Table S1. Primer sequences used for amplification of target genes in watermelon mosaic virus (WMV), melon, and zucchini plants.

Table S2. Common DEGs observed under the three temperature conditions highlighted in Fig. 3 for melon and zucchini varieties.

Table S3. DEGs from melon plant varieties under the combination of virus infection and termperature.

Table S4. DEGs from zucchini plant varieties under the combination of viral infection and temperature.

Table S5. DEGs from melon plant varieties under different temperature conditions.

Table S6. DEGs from zucchini plant varieties under different temperature conditions.

Table S7. GO enrichment analysis of melon plant varieties, which were specifically regulated by the combination of viral infection and temperature, shown in Fig. 4.

Table S8. GO enrichment analysis of zucchini plant varieties, which were specifically regulated by the combination of viral infection and temperature, shown in Fig. 5.
