# Supplementary material for: Thermotolerance elicits specific genes in cucurbit plants as a response to the combined effect of viral infection and temperature stress
Source: J Exp Bot. 2025 Jun 24;76(18):5305–19. doi: 10.1093/jxb/eraf277 (PMC12596120; doi:10.1093/jxb/eraf277)
Supplement: eraf277_Supplementary_Data [file eraf277_supplementary_data.zip › jexbot315331-file001.pdf]

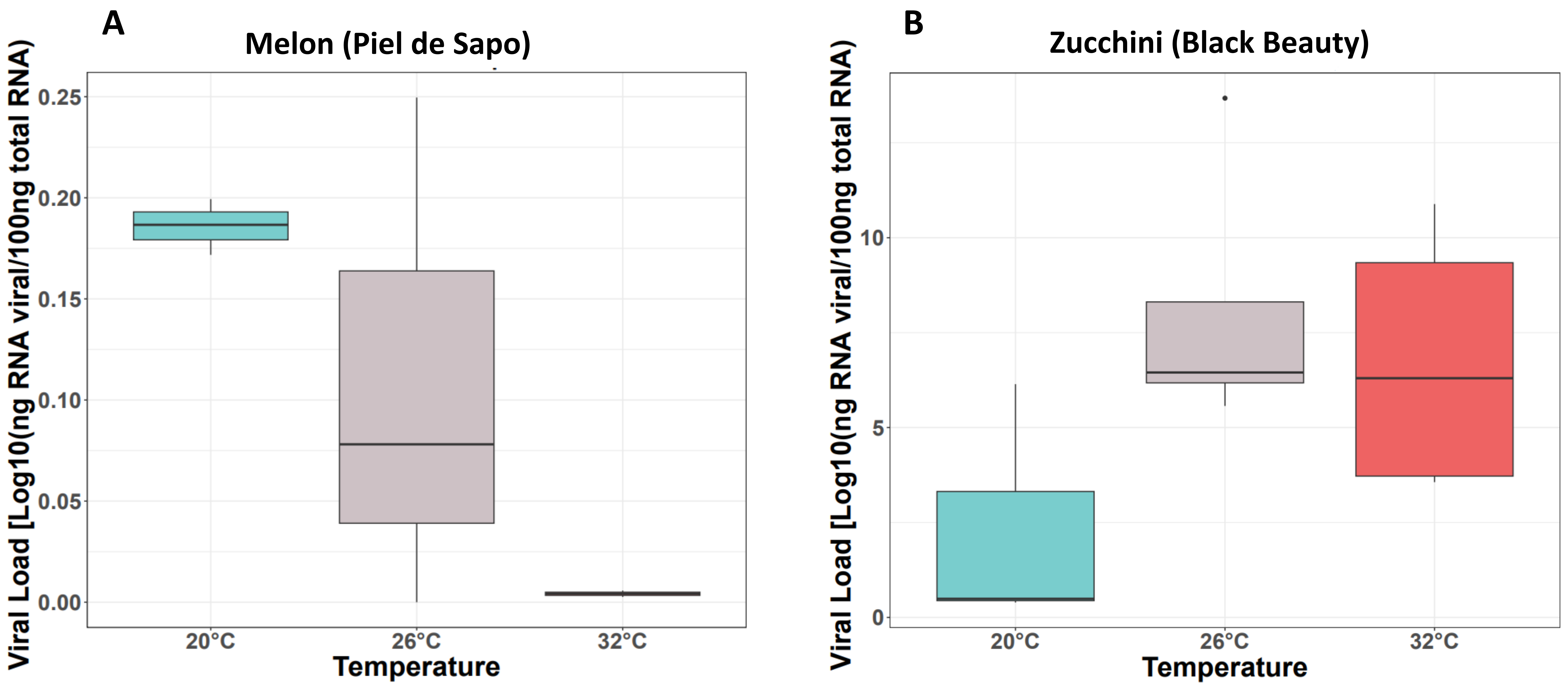

Figure S1. Viral load (mean and SE error bars, n = 3) of WMV infectious clones (MeWMV7) in commercial melon (Piel de sapo) (A) and zucchini (Black beauty) (B) plants at 30 dpi under three different growth temperatures: 20 °C/16 °C (Low, blue color), 26 °C/20 °C (Medium, grey color) or 32 °C/24 °C (High, red color). Viral RNA accumulation was determined using absolute quantitative RT-PCR. RNA transcripts of P1 were serially diluted (10-fold) to generate external standard curves. The RNA concentration in each sample (ng of viral RNA per 100 ng of total RNA) was estimated from the cycle threshold (Ct) values obtained from each independent biological assay, with three biological replicates at each time point.

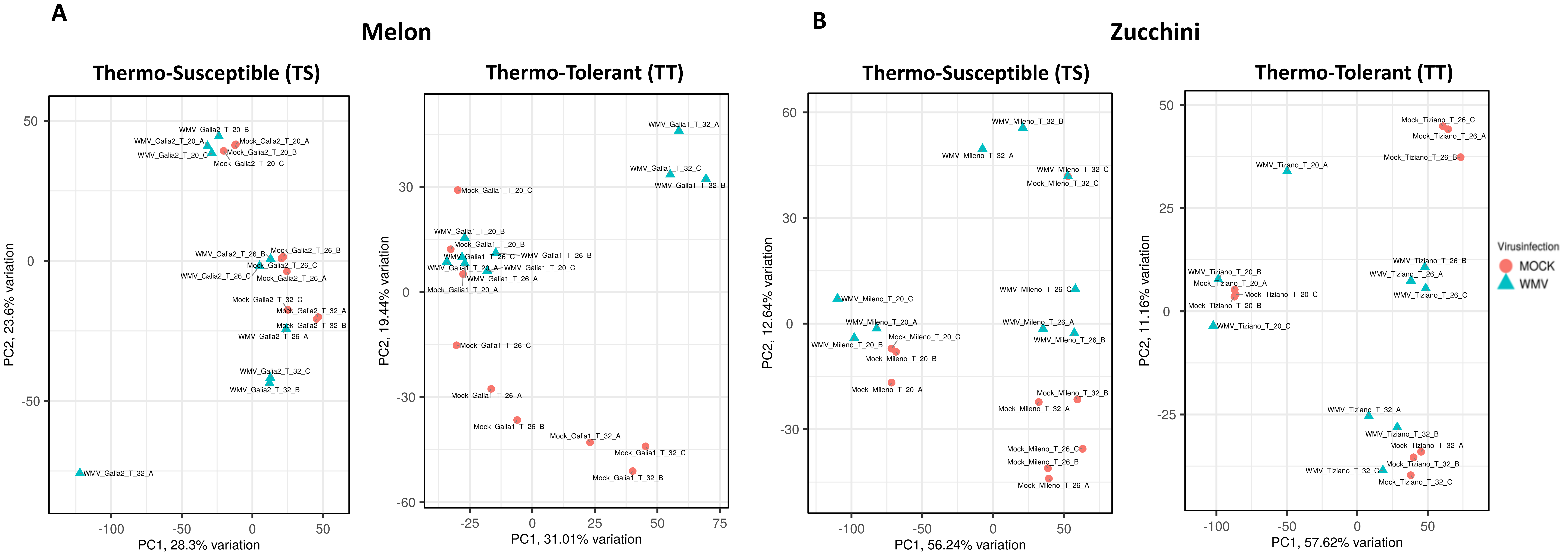

Figure S2. Principal component analysis (PCA) of thermosusceptible (TS) and thermotolerant (TT) melon **(A)** and zucchini **(B)** plants. Mock and infected samples were labeled with the corresponding temperature conditions (20, 26, and 32 °C), including replicates (A, B, and C), and represented by a red circle and a blue triangle, respectively. PCA was performed based on the read count data using iDEP 2.01.

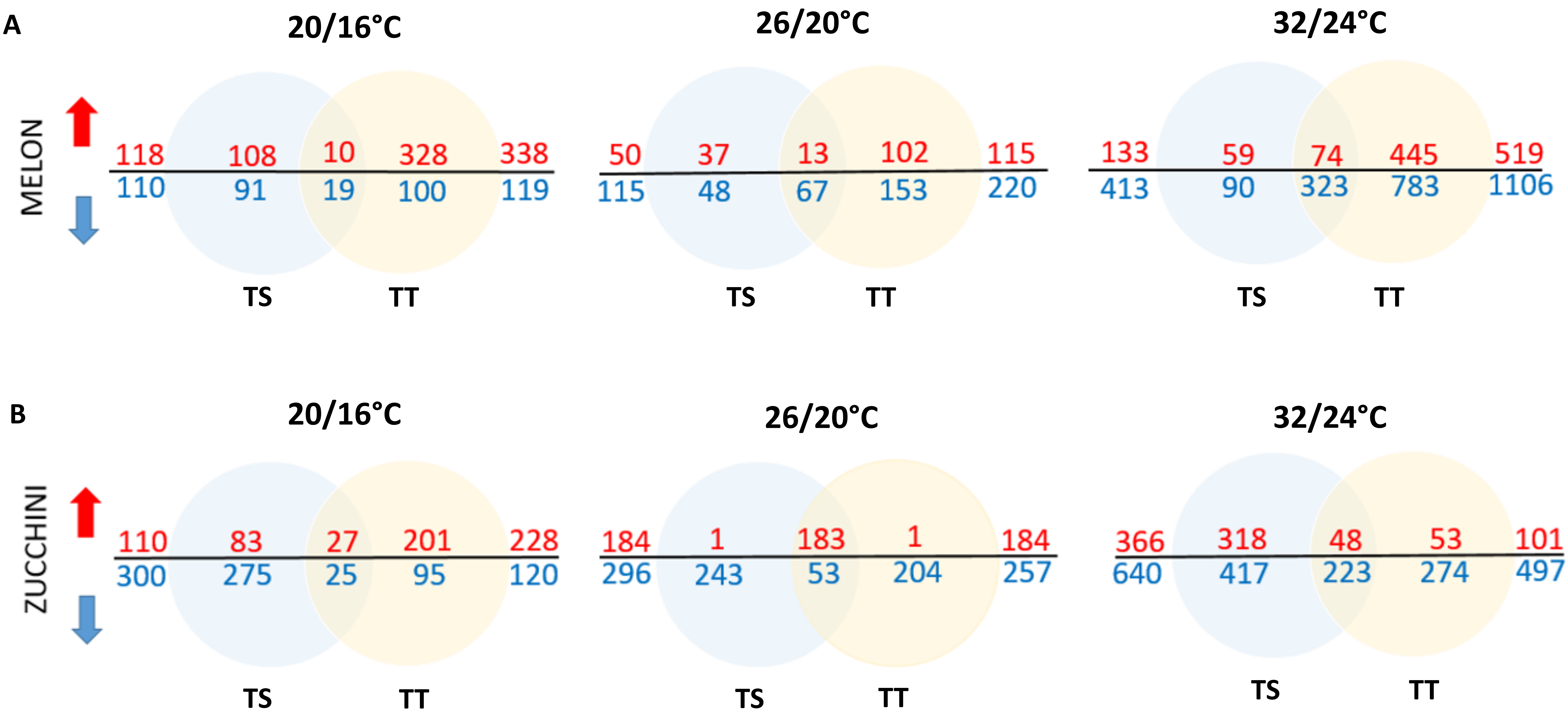

Figure S3. Venn diagrams of the overlap of upregulated and downregulated genes between thermotolerant (TT) and thermosusceptible (TS) **(A)** melon plants and between thermotolerant (TT) and thermosusceptible (TS) **(B)** zucchini plants under each stress treatment. Venn diagrams were constructed based on the genes listed in Tables S2 and S3 in R studio.

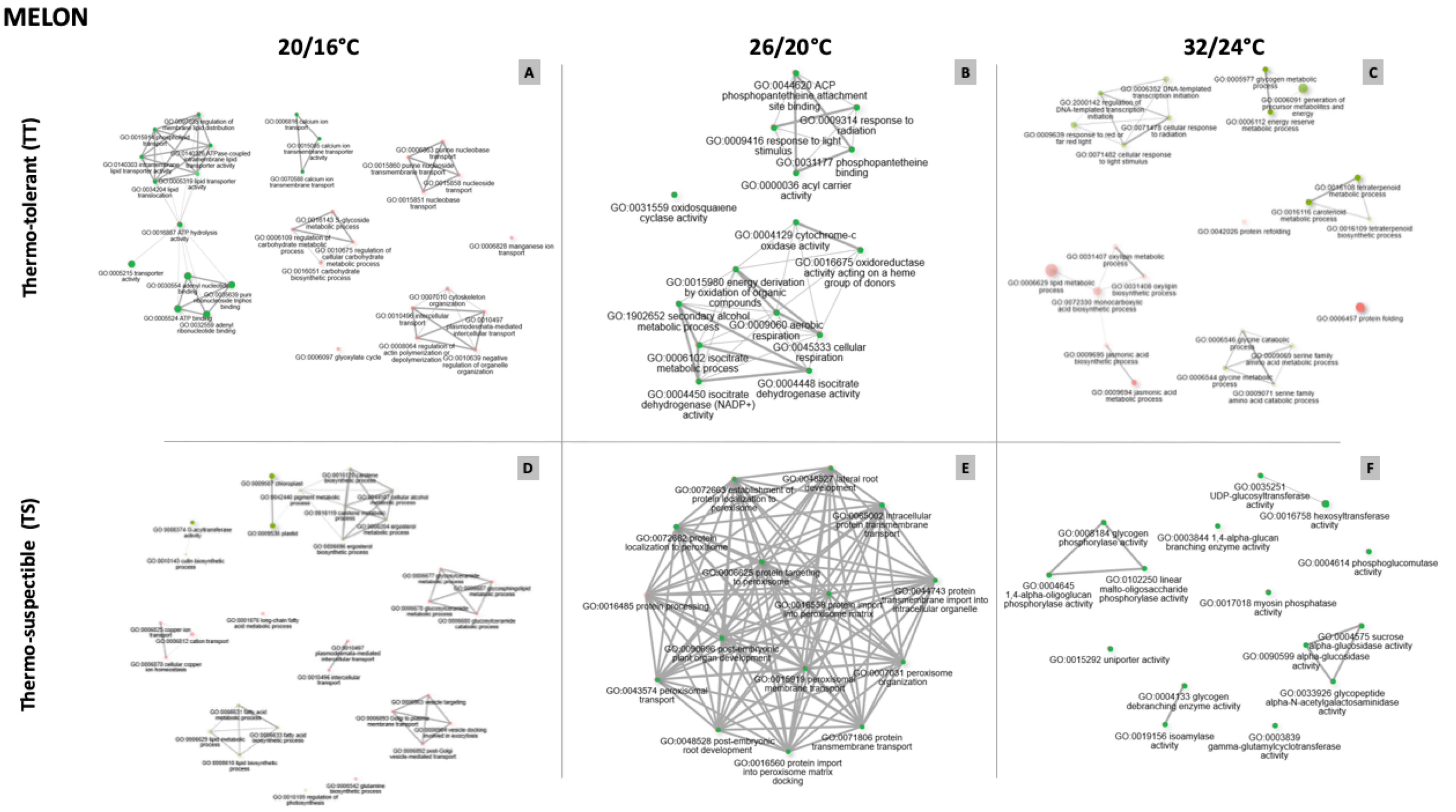

Figure S4. Pathway analysis and GO term networks based on molecular function and biological processes of specific DEGs under the combination of Temperature+WMV infection in melon plant varieties (FDR cutoff: 0.3). The nodes represent the enriched terms, and the size of the circles is proportional to the number of genes. The connections (or edges) between nodes indicated that they shared 30 % or more genes with similar molecular functions and biological processes. Thicker edges represent more overlapping genes, and green and red colors represent upregulated and downregulated genes, respectively. Networks are shown for thermotolerance (TT) at low (A), medium (B), and high (C) temperatures, and thermosusceptibility (TS) at low (D), medium (E), and high (F) temperatures. The raw data used in this figure are presented in Table S6.

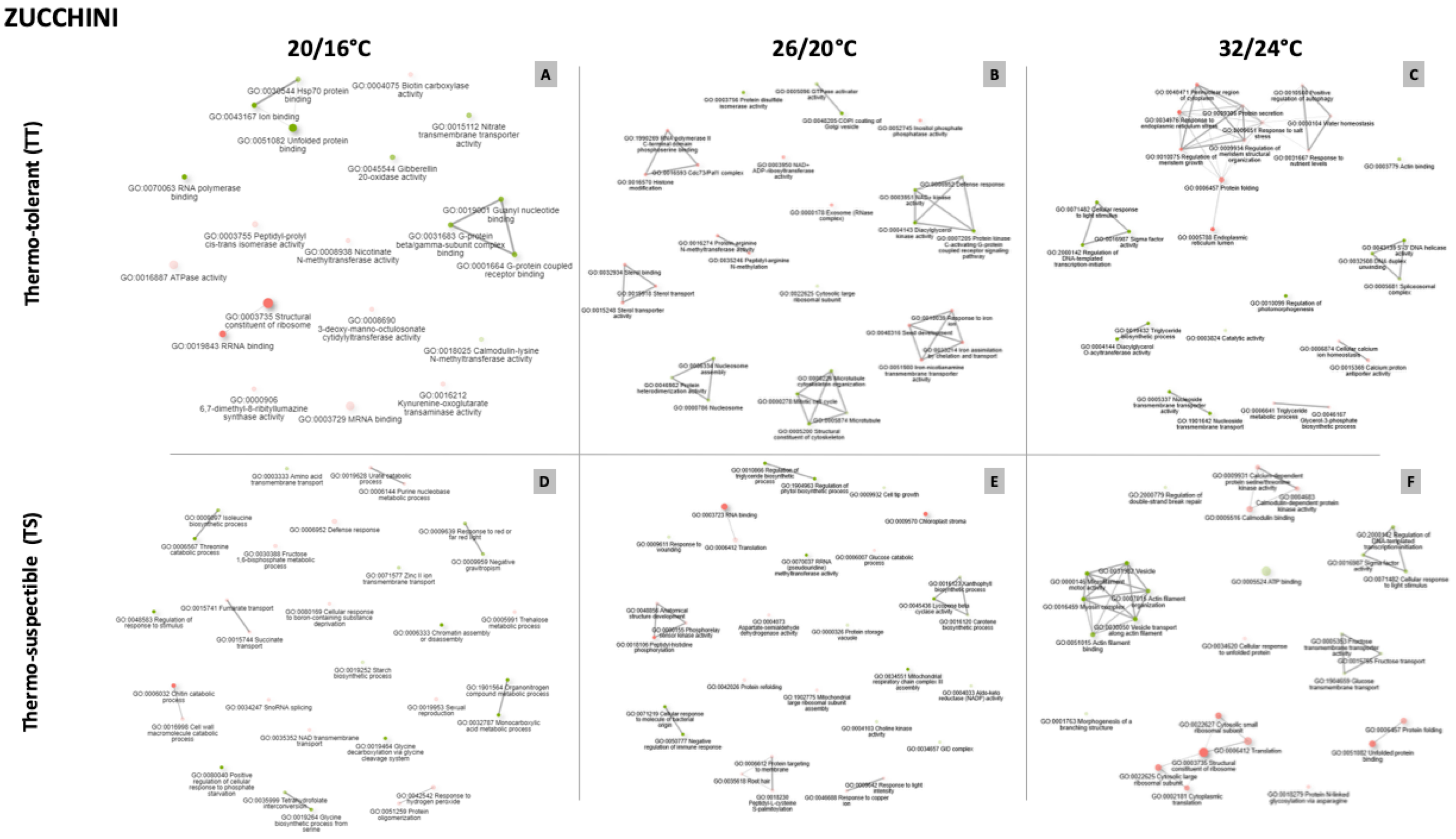

Figure S5. Pathway analysis and GO term networks based on molecular function and biological processes of specific DEGs under the combination of Temperature+WMV infection in zucchini plant varieties (FDR cutoff: 0.3). The nodes represent the enriched terms, and the size of the circles is proportional to the number of genes. The connections (or edges) between nodes indicated that they shared 30 % or more genes with similar molecular functions and biological processes. Thicker edges represent more overlapping genes. Green and red colors represent upregulated and downregulated genes, respectively. Networks are shown for thermotolerance (TT) at low (A), medium (B), and high (C) temperatures, and thermosusceptibility (TS) at low (D), medium (E), and high (F) temperatures. The raw data used in this figure are presented in Table S7.
